# Supplementary material for: Are carbon emissions trading and green financial instruments synergistic? -Comprehensive quantitative research based on content analysis
Source: PLoS One. 2024 Mar 7;19(3):e0298601. doi: 10.1371/journal.pone.0298601 (PMC10919601; doi:10.1371/journal.pone.0298601)
Supplement: S1 Data — (ZIP) [file pone.0298601.s001.zip › Data/地级市Z_F工作报告.zip!/╔┬╬≈/▒a╝a╩╨/└┤╫╘╣1⁄2╓┌║┼í╛┬φ┐╦╩2╛▌═°í┐_2017.docx]

各位代表：

现在，我代表市人民政府向大会作工作报告，请予审议，并请市政协委员和其他列席人员提出意见。

2016年和过去五年工作回顾

过去的一年，面对复杂严峻的经济形势，市政府坚持需求侧与供给侧两端发力，优存量与扩增量齐头并进，创新实干，主动作为，较好完成了市十四届人大六次会议确定的主要目标任务。全市实现地区生产总值1932.1亿元，增长9.3%；完成地方财政收入75.1亿元，同口径增长11%；城乡居民人均可支配收入达到31730元和10287元，分别增长7.7%、8.2%，实现了“十三五”稳健起步、良好开局。

调结构，产业培育成效显著。工业转型升级步伐加快，全面实施工业强市“1553”行动计划，吉利整车生产线建成投产，32户配套企业入园，90万吨甲醇、高锰钢辙叉生产基地等一批重点项目顺利开工，机器人智能制造产业园首批13个项目落地，清华紫光现代医药科技产业园建设进展顺利，和氏乳业建成亚洲最大的羊乳粉智能生产线，光伏、风力发电装机容量达到22.9万千瓦，航空航天、互联网等新兴产业加速成长。全市规模以上工业实现增加值887.8亿元，增长9.4%。农业特色产业进一步壮大，新发展苹果10.1万亩、猕猴桃3万亩，飞鹤关山4万只奶山羊标准化示范场建成，蜂产品检测中心、电子交易中心启用，秦宝肉牛、绿丰源成为农村三产融合新亮点。旅游产业人气火爆，全年接待国内外游客6384万人次，实现旅游综合收入439亿元。太白山成功晋级国家5A级景区，大水川创建为国家4A级景区，关山草原景区、青峰峡景区、太白鳌山滑雪场、凤县革命纪念馆开园，千亩荷塘、七星小镇等乡村旅游示范点建成运营。夜间经济持续繁荣，太阳市酒吧街、神农烧烤园运行良好。电子商务产业蓬勃发展，我市荣获“互联网+”2015年度中国网络零售最具活力城市殊荣，社会消费品零售总额达到702.1亿元，增长14.6%。

促投资，项目建设加速推进。完成全社会固定资产投资3199.8亿元，增长24.1%。大力开展“招商季”活动，全年共落实招商引资项目865个，其中投资10亿元以上项目23个，实现到位资金954亿元。全年350个市级重点项目完成投资664亿元，航天产业基地、高端装备石油产业园二期、新能源汽车产销基地等212个项目超额完成投资，宝麟铁路通车运营，总投资近300亿元的宝汉高速宝鸡段、宝平高速陇县至陕甘界、太凤高速、法汤高速绛帐至汤峪段开工建设，宝鸡机场通过国家评审。实施PPP项目65个，吸引社会资本80.9亿元，通过争取国家专项建设基金、设立投资引导基金和政府购买服务等方式，共为全市基础设施和民生项目建设募集各类资金近300亿元。

强基础，城市品位不断提升。编制完成《幸福宝鸡规划》，城市轻轨线网规划通过省级评审，我市列入省级海绵城市试点。植物园、联盟、阳平三座渭河大桥，以及虢磻路铁路立交桥、金陵一桥加固等工程开工建设，金台大道等16条城市道路改造提升全面完成，滨河路3处涵洞、宏文南路等建成通车，虢镇南环路等5条城市地下综合管廊建设进展顺利，新东岭国金中心主体建成，市文化艺术中心主体工程封顶。城市“五拆”工作成效显著，拆除各类建筑物13.3万平方米。金陵新村等5个片区老旧小区改造有序推进，政府购买棚改服务工作在全省率先启动，保障性住房建设位居全省前列。新发展天然气用户2.3万户，新增集中供热面积270万平方米，“光网宝鸡”建设任务全面完成。重点区域开发建设加力提速，科技新城道路管网建设步伐加快，高铁新城配套工程全面启动；蟠龙新区主干路网基本形成；蔡家坡入选国家新型城镇化综合试点，城市基础设施、教育中心等项目加快建设。实施县城改造提升项目325个，县城内涵和品质明显提高，汤峪镇跻身全国首批特色小镇。

抓改革，市场活力明显增强。政府机构职能转变和事业单位改革稳步推进，编制完成市县政府部门“六单一图”，取消下放行政审批事项217项，市级行政审批事项总数减少41%。积极推进商事制度改革，新增市场主体2.73万户。全面推行营改增，为企业减税降费8亿元。实施不动产统一登记，开展机动车驾驶证考试等改革试点，智慧城市建设全面启动。农村改革持续深化，土地承包经营权抵押贷款试点和国有林场改革顺利实施，土地承包经营权确权颁证工作基本完成。国企改革取得新进展，宝鸡纺织、医药集团公司挂牌成立。引进新设金融机构5家，宝鸡众力等5家企业成功挂牌“新三板”。协同创新加快推进，全省首次关中协同创新发展座谈会在我市召开，国家数控系统工程技术陕西分中心、激光加工国家工程研究西北区域中心、宝鸡科技协同创新西安中心启动运行，西北有色院宝鸡分院成立，宝鸡农业科技创新攻关同盟组建，国家油气钻井装备工程技术研究中心顺利通过验收评估。

重治理，生态环境持续改善。治污降霾强力推进，持续开展保卫蓝天“六大行动”，完成“燃气进村”5.4万户，市区空气质量二级及以上天数达到239天，连续两年位居关中各市前列。渭河治污提档升级，率先在全省实现了县城、重点镇生活污水处理设施全覆盖。深入开展大规模造林，完成关中大地园林化绿化57.1万亩、植树6200万株。大力实施城市增绿提质工程，成功创建为首批国家生态园林城市。渭河生态区建设步伐加快，编制了市区河流防洪与生态环境建设规划，城区段河道治理力度加大，完成渭河代家湾北岸绿化工程。美丽乡村建设持续加力，对630个行政村进行分类整治，实现全市农村环境综合整治全覆盖。

办实事，人民生活更加幸福。全市民生支出达到237亿元，占财政支出的83.8%。在西北地区率先实现国家义务教育发展基本均衡县和全省“双高双普”县全覆盖，推行了十三年免费教育，高考每万人二本上线率蝉联全省第一。机关事业单位养老保险制度全面实施，全民参保登记计划试点在省内率先完成，城乡居民养老保险“五个不出村”经办服务模式全省领先，被征地农民养老保险试点工作取得成功经验。就业创业扶持政策不断完善，城镇新增就业5万人，农村劳动力转移就业89万人。医改宝鸡模式得到国家肯定和推广，在全国率先建立免费基本生育制度，入选全国首批健康城市试点市。国家食品安全示范城市创建工作进展良好。在全省率先实现电梯责任保险全覆盖。农村低保保障标准达到扶贫标准，实施产业脱贫项目1995个，完成8949户易地扶贫搬迁任务，脱贫18.1万人。公共文化服务水平进一步提升，市、县“两馆一站”免费开放。全民健身活动蓬勃开展，成功举办15次全国大型赛事。全国双拥模范城创建实现“八连冠”，被评为全国人民防空先进城市，荣获中国西部社区养老示范城市称号。投资153亿元的“十件实事”全面完成，农村公路“最后一公里”建设完成1000公里，农村幸福院在全省率先实现中心村全覆盖。

各位代表，本届政府的五年是宝鸡发展历史上极不平凡的五年，我们顶住经济下行压力，克服转型升级带来的阵痛，攻坚克难，砥砺前行，谱写了宝鸡发展历程上的新篇章。

五年来，我们牢牢把握加快发展这个第一要务，抢抓新一轮西部大开发和关天规划实施的历史机遇，在政策对接中勇闯新路，在承接转移中转型升级，在产业培育中抢占高端。全市三次产业占比达到8.9∶63.5∶27.6，地区生产总值年均增速分别高于全国、全省4.2和1.9个百分点，规模以上工业增加值、地方财政收入、社会消费品零售总额、城乡居民人均可支配收入平均增速保持两位数增长，全市经济增速始终稳居全省第一方阵。

五年来，我们牢牢把握项目建设这个关键，千方百计扩大有效投资。累计引进亿元以上项目872个，实现到位资金3732亿元，比2011年翻了一番，吉利汽车、农夫山泉、青岛啤酒等14户世界500强、中国500强企业落户宝鸡。实施重点项目800多个，全社会固定资产投资是2011年的3.17倍。西宝客专、西宝和绛法高速改扩建等一批重大基础设施项目建成投用，60万吨甲醇、郭家河500万吨煤矿等一批重大产业项目竣工投产。

五年来，我们牢牢把握改革创新这个核心动力，破除体制机制障碍，发展活力进一步增强。行政审批制度、财税金融、管委会体制等改革不断深化，政府机构、公务用车等改革基本完成。以机器人关键零部件制造、旋翼机、无人机研发为代表的多项技术国内领先，大数据、新能源汽车等新兴业态从无到有，高端装备制造、新一代信息技术取得新突破。

五年来，我们牢牢把握民生改善这个根本目的，让改革发展成果更多惠及全市人民。每年把新增财力和财政支出“两个80%”用于民生，投资900亿元实施民生十大工程，八大创建结硕果、教育医疗惠民生、养老就业有保障、贫困人口稳脱贫、社区服务暖民心、农村环境展新颜，形成了一批全域覆盖、全省领先、全国有位的经验模式，社会治安满意率连续五年位居全省前列。

五年来，我们牢牢把握作风建设这个重要保障，转作风、促政风、带民风，提升政府工作的执行力和公信力。持续推行评议科长、干部代办、惠民事项公开等措施，政风行风测评群众满意度达到94.5%，稳居全省第一。深入开展“双千”活动，推动“三项机制”落实，精简文件、压缩会议、提高效率，政府工作人员主动担当、埋头苦干，以转作风的成效取信于民、推动发展。

同时，审计、统计、安监、气象、档案、粮食、物价、地震、地志、民族宗教、国家安全、妇女儿童、侨务外事、红十字会等工作也取得了新的成绩。

各位代表，回顾本届政府的五年，发展成绩来之不易，这是市委正确领导的结果，是市人大、市政协监督与支持的结果，饱含了各位代表、各位委员的智慧和力量，凝聚了全市人民的心血和汗水。在此，我代表市政府，向人大代表、政协委员、各民主党派、各人民团体、社会各界人士，向驻宝企事业单位、人民解放军、武警驻宝部队全体官兵、公安干警，表示崇高的敬意和诚挚的感谢!

在充分肯定成绩的同时，我们也清醒地看到，我市经济社会发展中还存在不少困难和问题：稳增长、调结构、产业转型升级任务艰巨，推进新动能成长和传统动能提升，还需要付出更大努力；县域经济实力不强、非公经济比重不大、现代服务业水平不高的问题还未得到根本性扭转，发展的整体性、协调性有待进一步增强；脱贫攻坚任务重、时间紧，群众就业、上学、看病等方面的难题仍然较多，大气污染治理压力依然较大；适应新常态的体制机制尚未完全建立，少数干部的工作作风和水平仍需改进提升。对此，我们将高度重视，认真研究，采取有力措施，切实加以解决。

今后五年的总体思路和目标任务

未来五年是宝鸡实现全面小康的决胜期，也是加快追赶超越的攻坚期。当前，我国经济进入速度换挡、结构调整、动力转换的新常态，经济发展的不确定性、不稳定性较大，但长期向好的基本面没有改变。站在新的历史起点审视宝鸡，“一带一路”、支持川陕革命老区振兴发展和关中城市群建设等重大战略机遇叠加，政策拉动和要素聚集效应将逐步显现；近年来我市经济持续快速发展，为进一步提升综合实力奠定了坚实基础、赢得了更多主动；广大干部群众谋求新发展、推动新跨越的氛围空前浓厚，为实现追赶超越提供了强大动力。只要我们把握机遇，主动作为，盯住目标追，朝着方向赶，撸起袖子加油干，就一定能够实现追赶超越，争先进位，开创宝鸡经济社会发展新局面。

按照市第十二次党代会总体部署，今后五年政府工作的总体要求是：深入贯彻习近平总书记系列重要讲话特别是来陕视察重要讲话精神，践行“五大发展理念”，按照省、市委决策部署，紧紧围绕全面建设最具幸福感城市总目标，以“不忘初心、追赶超越、群众满意”为主线，突出投资增效、工业强基、旅游带动、县域突破、脱贫攻坚五项重点，全面落实“十三五”经济社会发展规划，不断提升群众的富足感、舒适感、安全感、自豪感，把宝鸡打造成市强民富之城、生态宜居之城、法治安宁之城、人文和美之城，确保到2020年全面建成小康社会，基本建成关天副中心城市，向基本实现现代化迈进。

经济社会发展的主要任务是：经济发展质量效益显著提高，地区生产总值年均增速高于全省2个百分点左右，进入全国地级市经济总量前100位，经济结构持续优化，以“四基地一中心”为支撑的现代产业体系基本形成。人民生活品质大幅提升，城镇居民、农村居民人均可支配收入年均增长10%和11%以上，实现基本公共服务均等化。副中心城市地位进一步强化，对要素的吸纳能力和经济的辐射能力明显增强，形成关中西部百公里城镇群，城镇化率超过60%。生态环境质量显著改善，万元GDP综合能耗继续降低，森林覆盖率进一步提高，大气质量明显好转，力争率先建成国家生态文明建设示范区。

围绕实现上述目标任务，重点做好以下几方面工作：

——做强投资增效、开放招商主引擎。实施万亿项目带动计划，加快工业创新、现代农业、丝路商贸、文化旅游、交通枢纽、城镇设施、生态环保、公共服务等八大领域700个大项目建设，五年投资过万亿。打好招商引资“组合拳”，突出招大引强、产业招商，力争一批投资过百亿、产出过百亿的大项目落户宝鸡。打造全国高端石油装备、数控机床、特色农产品出口基地，构建西部内陆开放新高地。

——提升工业强基、创新转型竞争力。加快实施“1553”行动计划，推进工业强基“六大工程”，工业产值实现倍增，到2021年，全市建成汽车及零部件、能源化工等5个千亿集群，建成战略新兴产业和传统特色产业2个五百亿集群，成为全国高端装备制造业基地和国家级新材料、高技术产业基地。建设国家创新型城市，建成全省工业转型升级和提质增效先行示范区、人才发展改革试验区。推进十大重点领域体制改革落地见效，全面激发市场主体活力，持续释放改革红利。

——汇聚旅游带动、三产融合新势能。深入实施“旅游+”战略，加快构筑“一核一廊两带三区四组团”旅游发展格局，创建国家全域旅游示范市。到2021年全市建成5A级景区4个以上，年游客接待量突破1亿人次，旅游综合收入突破800亿元。大力发展互联网经济和总部型经济，加快发展大数据、研发设计等生产性服务业，支持发展商贸流通等生活性服务业，创建国家物流电商示范园，建成全国电子商务示范市，建设区域商贸物流中心和金融商务区。

——构建县域突破、协调发展大格局。加速园区集聚、产城融合，建成一批新型工业强县、文化旅游名县和特色农业大县。全面推进“一主三副”大城市建设，持续做美城市、做强县城、做大集镇，推动园区、景区、城区、社区协调互动、融合发展。打造西部交通新枢纽，实现关天经济区2小时通达，形成周边省会城市4小时经济圈。

——打赢精准脱贫、同步小康攻坚战。着力培育脱贫支柱产业，大力改善生产生活条件，加快推进扶贫移民搬迁，2019年贫困县全部摘帽，2020年稳定实现扶贫对象“两不愁、三保障”，让贫困群众迈向业兴、家富、人和、村美的新生活。

2017年的主要工作

今年，政府工作的基本要求是：坚持稳中求进主基调，把“稳”作为工作底线，把“进”作为发展要求，致力加压奋进，聚焦追赶超越，全面做好稳增长、促改革、调结构、惠民生、防风险各项工作，促进经济快速健康发展、社会和谐稳定。

主要预期目标是：地区生产总值增长10%左右，地方财政收入增长8%左右，全社会固定资产投资增长20%左右，社会消费品零售总额增长13%，城乡居民人均可支配收入分别增长10%左右，城镇登记失业率控制在3.9%以内，万元GDP能耗及主要污染物排放量完成省考目标。

重点做好七个方面的工作：

一、强力推进项目建设，夯实追赶超越基础

把项目建设作为追赶超越的第一动力，千方百计扩大有效投资，以大项目引领大发展、实现大跨越。

下好项目建设“先手棋”。实行项目“周策划、月收集、季推介”制度，推动项目建设接替有序、滚动发展。围绕中省政策抓谋划，准确把握国家稳增长、促投资政策动向，瞄准城市综合管廊、海绵城市建设、民生扶贫、产业发展和交通水利基础设施建设等方面，精心策划一批重点项目，争取中省资金不少于180亿元。围绕主导产业抓谋划，充分挖掘自身资源和产业优势，讲好“宝鸡故事”，在汽车产业配套、智能制造、能源化工、食品加工、文化旅游等方面，重点谋划一批填补行业空白、提升产业层次、引领转型发展的好项目，进一步做大做强特色优势产业。围绕创新发展抓谋划，借力科技创新，以补齐发展短板和适应新生活、新消费需求为核心，积极谋划一批两化融合、军民融合、协同创新和健康养老等成长性好的项目，支撑和引领未来发展。围绕PPP项目抓谋划，继续在市政设施、公共服务等领域推出一批PPP项目，全年至少开展2次推介签约活动，充分激发民间投资活力。

抓好招商引资“大会战”。继续开展“招商季”系列活动，实现到位资金增长15%以上。突出主攻方向精准招商，围绕工业强市“1553”行动计划、旅游“5+”战略、农村一二三产融合发展，继续组建招商小分队，分行业、分区域定向研究招商信息，及时、持续跟踪国内外知名企业、行业领军企业的投资方向和投资动态，做实招商地图，做精招商手册，做细招商方案，重点在工业领域着力引进一批拥有核心技术、占据价值高端、带动作用明显的好项目。瞄准前沿领域全员招商，紧跟大众创业、万众创新和“互联网+”等发展新趋势，落实招商引资奖励办法，推行领导干部“三三制”，压实部门单位的招商任务，动员全社会参与招商，积极引进一批众创、众包、众扶、众筹平台，吸引天使、创业、产业投资基金，力争一批新技术、新产品、新业态项目落户宝鸡。聚焦追赶超越招大引强，对标发达地区和先进地市，跳出“城墙”思维，以更宽的眼界、更高的标准，提升招商引资工作水平。以招引工业项目为主，年内全市新引进3-5个投资过30亿元的大项目，高新区、蔡家坡经开区、蟠龙新区各引进2个以上投资过20亿元的产业项目，各县区至少引进1个过10亿元的项目，筑牢加快发展的根基。

打造西部投资“新洼地”。全力营造一流的发展环境，努力做到优质服务“零距离”、项目建设“零干扰”，推动项目落地生根、开工建设。破解服务瓶颈，继续落实“五个一”工作机制，大力推行“一线工作法”和“现场办公制”，及时高效解决实际困难和问题，用干部的辛劳换来发展的提速。破解资金瓶颈，强化财政改革，不断创新投融资机制，抓好政府投资引导基金运营，充分发挥子基金、投资担保公司的作用，确保重大基础设施、民生、生态项目建设和重大产业项目支持的资金供给。筹建消费金融公司，实现宝鸡农商银行、恒丰银行宝鸡分行开业运营，年内金融机构新增贷款余额130亿元以上，社会融资规模存量超过1800亿元。破解保障瓶颈，推行依法征拆、阳光征拆，严格效能问责，严肃查处恶意阻挠项目建设的违纪违法行为，解决项目开工难、建设难问题。实施追赶超越八大项目包计划，建设重点项目456个，确保完成年度投资965亿元。

二、聚力推进转型升级，加快工业强市建设

深入实施工业强市“1553”行动计划，推动优势支柱产业上档次、扩规模，战略新兴产业抓机遇、抢先机，传统特色产业抓提升、促转型，不断做大工业总量，提升发展质量和效益。

推进工业强基“六大工程”。实施汽车及零部件产业跨越工程，加快吉利发动机、汽车配套零部件、华山车辆纯电动中轻卡等项目建设，开工建设法士特中轻卡及新能源变速器项目，支持陕汽做大做强，确保吉利汽车达产达效，延伸汽车及零部件产业链条。实施钛及钛合金产业突破工程，抓好钛合金高端应用、钛合金骨科植入物、核级锆材生产、钛谷新材料检测中心等项目，推动产品升级，让钛产品迈向高端、步入生活。实施烟酒食品产业提升工程，扩大青啤宝鸡工业园生产能力，推进西凤酒城、今麦郎饮品、海升果蔬汁、眉县猕猴桃、陇县核桃深加工等项目建设，带动易拉罐、包装等配套企业成长，壮大烟酒食品产业规模。实施能源化工产业转型工程，推进90万吨甲醇、麟游新型煤化循环经济工业园和低热值发电等项目，力争北京神雾集团投资250亿元的80万吨聚乙烯多联产项目落户宝鸡，促进煤炭资源深度转化。实施优势装备制造产业创新工程，开工建设中铁电气化宝鸡科技产业园，加快高锰钢辙叉生产基地、中车时代扩能提升等项目建设，做强高端装备制造产业。实施战略新兴产业壮大工程，加快7107厂航天产业基地、光纤陀螺生产线、旋翼机产业园建设，引领航空航天产业占高端；加快凤县动力电池负极材料、宝鸡建筑节能产业园和光伏、风力发电等项目建设，推进新能源及节能环保产业扩规模；加快烽火通信车智能机电、金台大数据产业园二期、渭滨互联网大数据产业孵化基地等项目建设，实现电子信息及光通信产业新突破；加快紫光医药科技产业园、扶风生物科技产业园、西山“秦岭药谷”等项目建设，加速生物医药与健康设备产业快发展；加快推进数控机床智能化生产系统、机器人及智能制造产业园等项目，实施传统工业智能化改造，推动高档数控机床和智能制造产业上水平。

提高科技协同创新能力。加快建设国家创新型城市，实施科技创新“十大工程”，推动人才链、创新链、资本链、产业链深度融合。建设国家级、省级研发中心及分中心10个以上，支持高新区联合清华大学等高校建设创新研发中心，力争在北京、上海等地建立协同创新平台，谋划境外产业对口合作平台建设。落实人才引进政策，年内引进高端科技创新领军人才团队10个以上，培育创新型企业20户以上。加大政银企三方合作力度，推进年度投资143亿元的技改项目96个，把科技扶持资金和产业引导基金向重点技改项目倾斜，加快培育壮大新动能。以绿色低碳为方向，加快眉县新型防水材料、千阳海螺固体废弃物处置、岐山普宁服装产业园、凤县电解铅技改等项目建设，改造提升传统动能。

持续激发市场主体活力。落实“三去一降一补”政策，着力推进国企改革，加快西凤酒上市融资，启动宝纺集团产业整合搬迁技改，引进战略投资者参与宝鸡医药集团整合重组，增强国有企业竞争力。大力发展非公经济，构建“亲”“清”新型政商关系，实施百户中小工业企业培育成长工程，年内培育规上企业50户以上。充分发挥6个专项工作组和2个专家组作用，不断提高服务工业经济的能力和水平。抓好智慧工业园区建设，年内新增标准化厂房81万平方米。优化企业生产要素供给，加强用地保障，扩大直供电范围，启动国有企业“三供一业”分离移交，加快剥离国有企业办社会职能，为企业卸包袱、减负担、解难题。

三、着力推进全域旅游，带动三产快速发展

坚持把服务业作为补短板、调结构的重要环节，做火旅游、做旺商贸、促进消费，持续提升第三产业整体实力。

推进全域旅游，做靓旅游品牌。以品质旅游为统领，大力实施旅游“5+”战略，创建国家全域旅游示范市。建设精品景区，围绕打造周秦文化、佛骨圣地、秦岭山水、西府民俗四大品牌，加快建设岐山周文化景区，启动建设周原遗址公园，打造国家记忆工程；实施大美佛汤城项目，推进“世界佛都”建设，提升法门寺佛文化景区影响力；支持太极源景区、龙门洞景区建设，彰显道文化特色。加大太白山创建国际旅游度假区和国家文化产业示范区建设力度，抓好鳌山滑雪场等项目，发展冰雪运动，打造秦岭山水旅游核心区；积极推进关山草原、青峰峡5A级景区创建，加快大水川、鸡峰山、黄柏塬、紫柏山等景区开发，抓好六川河西普陀旅游度假区、千湖国家湿地公园、大东湖景区等项目建设，让生态山水成为宝鸡旅游的靓丽名片。加快茵香水镇、美阳小镇、固关古街、凤翔酒街、岐山臊子面文化产业园等示范点建设，让游客体验“舌尖上的宝鸡”。坚持“赛事兴旅”，吸引重大赛事来宝举办，争取国内常设赛事落户我市。优化设施配套，实施道路畅通、旅游标识、厕所革命“三大行动”，完善沿线建筑、生态环境等旅游要素，建立自助导游体系，加快市、县两级游客服务中心和全市旅游数据中心建设，构建互联互通的全域旅游体系，让宝鸡旅游成为轻松之旅、快乐之旅。提升服务水平，加快旅游体制机制创新，组建市旅游发展委员会，建立与全域旅游相配套的公安、工商、法院等执法服务体系，推进旅游标准化管理。强化全市整体旅游形象宣传，开展多元化、立体式的宣传营销活动。全年接待游客突破7350万人次，实现旅游综合收入510亿元。

强化有效供给，释放消费潜能。围绕消费需求，培育新供给、新动力，实现社会消费品零售总额793.4亿元。完善商贸流通体系，持续提升经二路、金台大道、高新大道等商圈基础设施，确保银泰购物中心、千禧时代等项目建成开业。拓展社区家政、购物等服务业态，推进放心肉、放心菜、放心早餐等社区商业体系建设，打造15分钟便民生活服务圈，推动商贸流通业提质增效。大力发展新兴服务业态，积极创建国家电子商务示范城市，组建宝鸡电商联盟，培育电商龙头企业，实施“快递下乡”工程，实现线上线下融合发展、中心村电商全覆盖，让宝鸡的产品网走四方。加快发展研发设计、信息技术服务、金融等生产性服务业，大力发展创客空间、各类孵化器，创建宝鸡工业云平台，推动新兴服务业加速发展。持续繁荣夜间经济，推进石鼓文化商业项目，完成西府老街、封神演义文化主题游乐园等项目建设，做大规模、做旺人气、做亮品牌。

扩大对外贸易，提升开放水平。主动融入国家“一带一路”战略，打造丝绸之路经济带战略新枢纽。构建对外开放平台，启动宝鸡传化物流公路港项目，开工建设阳平铁路物流基地，加快推进宝鸡综合保税区、泰华物流产业园建设，形成丝绸之路经济带物流大通道。扩大外贸出口规模，推进常兴纺织产业园、蔡家坡高端装备产业园等外向型园区建设，建立检验检测、外贸孵化等公共服务平台，实施20户外贸企业技术改造项目，推动宝鸡跨境电商交易平台出口创汇实现新突破。加快“海外宝鸡”建设，支持宝光集团、宝鸡机床、红旗民爆等企业，在境外投资办厂或承包工程，带动成套设备、技术和人员输出。依托境外投资项目，在吉尔吉斯斯坦规划建设宝鸡工业园，带动我市更多的机械、建材、新型材料等优势企业走出去开拓市场。

四、全力推进脱贫攻坚，提升三农工作水平

围绕打赢脱贫攻坚战，以农业供给侧结构性改革带动农民增收，坚持强农惠农富农政策不减弱，促进农业强、农民富、农村美。

发展现代农业，推动脱贫致富。进一步完善全市农业产业结构调整区域化、特色化布局方案，着力提升粮经比、粮畜比、粮加比三个比例，加快发展县域特色经济，持续增强产业对脱贫增收的支撑能力。做好“三绿”文章，发展绿色种植，年内新发展优质苹果4万亩、猕猴桃3万亩、鲜杂果3万亩、核桃6.3万亩、苗木花卉3万亩，新建设施蔬菜基地5000亩。发展绿色养殖，以千阳、陇县为重点，建设30万只奶山羊基地，加快形成全国最大的奶山羊产业聚集区；支持石羊集团实施百万头生猪和百万只蛋鸡项目；加快建设秦岭蜂文化休闲园，发展中蜂示范基地5个、标准化养蜂示范场100个，打造农业经济新的增长点。发展绿色加工，在全市重点规划建设5个左右食品工业园，每个县区都实施一批农产品加工项目。实施“三品”工程，推进品种改良，抓好汇源高端苹果苗木基地、扶风县生猪云养殖示范项目建设，积极引进和推广优良品种，加快品种更新换代步伐；推进品质提升，大力推行生产标准化，完善农产品质量安全追溯体系，增加高质量农产品有效供给；推进品牌建设，组建宝鸡苹果产业联盟，举办世界猕猴桃研讨会，在一线城市新建宝鸡果品品牌店10个，在主销区举办宝鸡农产品专题推介活动，打响宝鸡苹果和蜂蜜、眉县猕猴桃三个区域公用品牌。推进“三产”融合，加快“农工商、产供销”一体化发展，培育新型农业经营主体，抓好益可达秦川牛、凤县农产品物流园等项目建设，启动建设宝鸡农业科技协同创新示范区和岐山国家农业公园，加快太白县国家级农业示范区创建步伐，支持陈仓区、岐山县创建国家农业可持续发展试验示范区，建成省级现代农业园区2个、市级10个，创建市级农民合作示范社30个，全产业链培育职业农民。支持陇县、千阳、太白启动绿色发展示范县建设，加快形成绿色生态产业链。

释放政策红利，助力脱贫攻坚。将民生项目、惠民政策最大限度地向贫困地区倾斜，加快推进移民搬迁工程，全年完成5257户18765人搬迁任务。结合天然林保护等重大生态工程，让农民既受益生态政策补贴，又通过就业实现增收。用好教育免费、教育资助、扶贫培训等优惠政策，斩断贫困代际传递。全面落实社保兜底政策，建立民政救助、医疗救助与精准扶贫无缝对接机制，不让一个贫困群众掉队。年内实现12.2万农村贫困人口脱贫，466个贫困村脱贫退出。

改善农村条件，夯实脱贫基础。大力推进高标准基本农田建设，发展节水灌溉10万亩，治理水土流失400平方公里。实施千河千阳段、嘉陵江凤县段、渭河陈仓上游段等防洪治理工程，加快麟游崔木、太白石沟水库建设进度，力争陇县南峡沟、眉县远门河、太白县龙王河等水库开工建设。实施新一轮农村电网改造升级工程，抓好县乡公路、危桥改造等项目。持续推进村史馆、家风馆建设。加强土壤污染防治和农村面源污染治理。建立长效管理机制，巩固提升农村环境综合整治成果，每年创建50个市级美丽乡村、100个县级美丽乡村。

五、致力推进城市建设，增强辐射带动能力

以新型城镇化为方向，加快建设副中心城市，提高城镇品质，不断提升城市的吸引力、承载力和竞争力。

突出规划引领，彰显城市特色风貌。加快编制新一轮《城市总体规划》，按照“一主三副一带两翼”市域空间布局，完善重点地段控制性详规，促进“多规合一”。严格执行《幸福宝鸡规划》，加强对渭河两岸、秦岭浅山区等重要区域城市风貌和建筑特色设计，实施城市“双修”工程，不断优化城市功能和景观风貌。改革规划管理，强化执行力和约束力，让城市发展更守规矩。

完善综合体系，强化中心城区功能。加强交通网络建设，加快市区五座大桥和城市轻轨建设，贯通新福二路与经二路，拓宽改造金陵东路，开展陈仓、千渭、钛谷、团结渭河大桥及南绕城高速辅道、引渭渠南路建设前期工作，缓解主城区交通拥堵。加速推进宝汉高速、太凤高速、连霍高速过境线、宝平高速陇县至陕甘界及10条干线公路建设，启动西安至法门寺城际铁路、合凤高速、眉太高速公路建设，加快关中环线宝鸡段、麟游至法门寺高速公路前期论证工作，力争宝鸡机场上半年立项，确保宝兰客专年内建成通车，巩固提升我市交通枢纽地位。加强配套设施建设，扎实开展海绵城市省级试点，推进川陕路、姜谭路等综合管廊建设，改造曙光路、东风路、新建路等雨污管网，加快旧城改造步伐，继续推进城市“五拆”工作。实施城市供水、供气、供热设施配套完善工程，开工建设宝二电向市区供热、麟游至宝鸡和陇千段天然气输气管道工程等项目，启动生活垃圾综合处理PPP项目，切实提高城市保障能力和宜居水平。加强城市景观建设，巩固提升国家生态园林城市创建成果，启动“城市绿荫”三年行动计划，加快千河立交景观绿岛、虢镇北坡森林公园和渭河城区段4个亲水平台建设，抓好蟠龙桥“一河两岸”亮化等工程，开展立体绿化、楼顶绿化试点，让市民开窗见绿、出门见景。

推进产城融合，提速重点区域开发。继续做强高新区，全面加快科技新城、高铁新城开发建设步伐，做大做强汽车及零部件、新材料、智能制造等优势产业，强化社会治理，着力打造创新发展示范区。加快重塑蔡家坡，围绕建设汽车产业聚集区、幸福美丽小城市的目标，突出中、轻、微、特、新及汽配六大产业体系，加快推进通家生产线改造、陕汽中轻卡产业基地、百万平方米标准化厂房、基础设施等项目建设，打响汽车新城名片。提速建设新蟠龙，高标准建设新区配套设施，培育新型、绿色产业体系，抓好西安交大蟠龙医院、长安大学兴华学院、蟠龙国际旅游度假区、蟠龙湖千亩生态休闲公园等项目建设，推动生态新城建设取得新进展。

加大支持力度，完善全域城镇体系。高点定位建设县城，推进凤翔撤县设区、眉县撤县设市工作，在建设新型城镇示范区上有新作为。加快陇县绿色食品工业园、秦源文化产业园、交通物流园建设，在打造生态新城区上有新进展。支持太白、凤县城区按照5A级景区标准规划建设，在全域高水平建设上有新跨越。加大岐山、扶风县城改造力度，在产城融合发展上有新突破。加强千阳、麟游县城综合基础设施建设，在增强承载能力上有新提升。年内，每个县区重点抓好1-2条街区及城区出入口的提升改造。突出特色做大城镇，支持国家重点镇、省级重点示范镇、文化旅游街区和3个市级重点镇建设，抓好80个特色镇改造提升，实施功能完善、品位提升、精细管理三大工程，带动农民就近城镇化，推动关中西部百公里城镇群加速崛起。

创新治理方式，提升城市管理水平。推进城市执法体制改革，整合城市管理相关职能，组建城市管理执法局，完善城市管理市场化运营模式，推动城市管理向城市治理转变，促进城市高效有序运行。围绕群众关心的交通拥堵、管理粗放、应急滞后等问题，下硬茬、出实招，有效缓解“城市病”。大力推广垃圾分类试点和清洁收运，确保餐厨废弃物资源化利用和无害化处理项目建成投用。深入开展市容市貌整治，加大背街小巷、城乡结合部、农贸市场等重点区域治理力度，城区道路机扫实现全覆盖。强化协调协同，推动政府、社会、市民同心同向行动，实现城市共治共管、共建共享。

六、努力推进生态建设，持续优化人居环境

把生态文明建设摆在更加突出的战略位置，坚持绿色、循环、低碳发展，努力打造天蓝、地绿、水清的美丽宝鸡。

建设绿色精品工程。以“生态大花园”统筹绿色宝鸡建设，突出城市出入口、城郊森林公园等重要节点，打造一批绿化精品景观和市民休闲林。以道路、河流为重点，抓好缺林少绿和断档区域绿化，完成连霍高速、宝平高速、西宝客专及干线公路沿线绿化提升，打造千里绿色长廊。推进河流湿地生态景观化，建设金陵河湿地公园，争取凤翔雍城湖列入国家湿地公园试点。开展森林城镇建设，全年完成绿化35万亩、植树4000万株，顺利通过关中大地园林化验收。

铁腕推进治污降霾。实行最严格的环保制度，全面落实源头严防、过程严管、质量严考、后果严惩的“四严”措施，重拳治霾，利剑斩污，努力推动治污降霾向科学施策、精准治理、主动治理转变。不断深化“6511”治霾模式，大力开展散煤整治、清洁能源、扬尘治理、工业减排、尾气达标、面源管控、应急响应、环境执法八大行动，突出抓好建筑工地扬尘、露天烧烤和小企业治理，推进电能替代，坚决淘汰1535辆“黄标车”，加强预测预报和预警会商，推进应急联动和联合执法，不断改善空气质量。

持续抓好渭河治理。以渭河市区段生态治理和“百里画廊”生态提升为重点，按照市区河道“滩区治理全覆盖、堤顶道路硬化全贯通、一河两岸绿化全提升”目标，加快推进团结运动公园、姜谭公园、渭河北岸自行车专用道等27个重点项目，实施眉县、扶风、岐山等渭河区段生态修复及湿地建设提升工程，打造水景相望、运动休闲、产业集聚的立体生态走廊。推行河长制，实施渭河支流截污和水体污染补偿考核，完成蔡家坡污水处理厂、高新区污水处理厂二期扩建和陈仓区污水处理厂、扶风县百合污水处理厂提标改造工程，确保渭河、小韦河、嘉陵江等出境水质稳定达标，真正把渭河建成宝鸡人民的生态河、休闲河、景观河。

七、倾力推进民生建设，提高群众幸福指数

坚持以人民为中心，把更多的发展成果转化为惠民实效，让发展更有温度、让幸福更有质感。

加强社会保障为民利民。大力推进大众创业，实施高校毕业生就业促进、大学生创业引领、农民工返乡创业等行动计划，加强就业服务保障，年内新增市级创业孵化基地5个，实现城镇新增就业4.5万人，转移农村劳动力85万人。巩固提升“大社保”模式，深化全民参保登记计划成果，建立健全统一的城乡居民基本医疗保险制度。积极探索建立城乡居民基础养老金正常增长机制，逐步推行被征地农民参加城镇企业职工基本养老保险，推进新农合市级统筹，完善城乡居民基本养老保险“五个不出村”服务模式。不断强化养老助残保障，大力推广“智慧养老”模式，抓好扶风社会福利中心建设和凤县社会福利中心改扩建，完成市殡仪馆设施提升改造项目，建成农村公益性公墓70个。规范农村幸福院管理运行，建立“空巢老人”关爱帮扶机制，落实留守儿童、残疾人关爱政策，让弱势群体感受到更多温暖。

发展社会事业便民惠民。优先发展教育事业，持续深化中小学德育行动、普优行动和家庭教育“绿荫计划”，加强4个城乡教育联盟建设，加快教育现代化先进县区创建。支持宝鸡文理学院筹建宝鸡大学。推动宝鸡职业技术学院建设省内一流、全国知名的高职院校，开工建设市公共实训中心，打响“宝鸡技工”品牌。提升医疗服务水平，深化公立医院及医疗服务价格改革，全面推行药品和医用耗材“两票制”，健全分级诊疗制度，巩固提升医改宝鸡模式。大力推进健康城市建设“十大行动”，加快市第三人民医院养老康复中心、市中医院中医药养老示范基地、麟游九成宫怡心谷养生基地建设，吸引社会资本发展健康服务产业。优化全面二孩政策实施环境，落实计生奖扶政策，促进人口协调发展。丰富群众文化生活，持续开展群众性文化体育活动，承办金砖国家国际象棋大师赛、全国青年跳水冠军赛、全国第三届太极拳邀请赛，办好市第十一届运动会、第九届全市体育健身大会等重大赛事。实施文化惠民工程，加快市文化艺术中心建设，支持有条件的县区推出品牌演艺节目，不断增强群众的获得感。

创新社会治理安民亲民。加强基础制度建设和社会信用管理，推进社会治理精细化、信息化、网格化，构建全民共建共享的社会治理格局。实施“七五”普法，推动全社会特别是公职人员尊法学法守法用法。扎实推进最安全城市建设，依法惩治各类违法犯罪行为，继续保持“两率一度”全省领先。完善社会稳定风险评估和矛盾纠纷调处化解机制，推进法治信访和“三无”县区创建。深入开展国家食品安全示范城市创建，确保各项工作按期达标。落实安全生产责任，强化源头管理，抓好重点领域监管，有效预防和遏制重特大安全事故发生。

各位代表，群众利益无小事，人民对美好生活的向往就是政府的奋斗目标，今年我们将继续为群众办好十件实事：一是补助投资10亿元，新建通村公路3500公里，全面完成农村公路“最后一公里”建设任务。二是投资4000万元，实施农村饮水安全巩固提升工程，改善6万群众饮水条件。三是投资5亿元，在市区新建公共停车位6000个，进一步缓解“停车难”问题。四是投资3000万元，实施技能培训全覆盖惠民工程，免费就业创业培训3万人。五是实施324个农民健身工程，让体育惠民政策走近群众。六是投资1.2亿元，继续实施“燃气进村”项目，让3万户群众用上清洁能源。七是投资31亿元，持续推进棚户区和老旧小区改造，让居民生活更方便、更舒心。八是投资2227万元，启动10个社区日间照料中心建设，完成17个城镇社区基础设施提升和52个农村社区服务中心建设项目。九是市区和县城学校操场、体育场馆免费开放，让群众有更多的健身场所。十是开展“政府出资、剧团送戏、群众受惠”演出活动，覆盖全市每个乡镇，将文化大餐送到群众家门口。

切实加强政府自身建设

新形势赋予新使命，新常态要有新作为。新一届政府将努力践行“加压奋进、追赶超越、创新实干”的工作理念，一心一意谋发展，聚精会神搞建设，创造无愧于时代、无愧于人民的新业绩。

建设锐意创新政府。牢固树立追赶超越理念，对标先进，聚焦短板，打破思维定势，突破路径依赖，创新思路方法，以思想大解放促进经济大发展。统筹推进各领域重点改革。推动“放管服”改革向纵深发展，进一步减程序、减费用、减时限，以行政权力大“瘦身”为市场主体添活力。加强干部理论武装和专业知识培训，在政府系统深入开展《干部经济知识简明读本》学习活动，补齐本领恐慌、能力不足短板，让各级干部成为经济转型发展的“推动者”和“操盘手”。积极借助智库外脑，充分发挥市决策咨询委员会作用，注重全要素聚集整合，坚持运用市场手段破解发展瓶颈制约，提高做好经济工作的专业化、科学化水平。

建设实干担当政府。认真贯彻落实“三项机制”，健全完善实施细则，理直气壮地支持善谋勇为、敢于担当者，不断激发干事创业热情。各县区、各部门要高起点制定追赶超越目标，建立项目清单制度、追赶超越季度点评制度，层层分解任务、压实担子。开展“十强镇”“百强村”评选活动，推动县域经济争先进位，力争每个县区在全省的排名位次都有前移，巩固提升凤县在全省十强县中的位次，争取渭滨区重返五强区。全体政府组成人员要敢于负责、主动作为，对重点项目、重大事项、重要工作，快谋划、快上手、快实施。要发扬“钉钉子”精神和“马上就办”的作风，一项任务一项任务完成，一个项目一个项目落地，一个节点一个节点推进，增强执行力，提高战斗力。

建设为民服务政府。围绕建设“西部一流、全国领先”智慧宝鸡战略定位，全面启动“12345”智慧城市建设工程，建立一个大数据中心、两个云平台、整合三类基础资源、四类行业智慧应用和五个通用应用，利用信息化技术，更好地服务企业、服务群众、服务社会，让群众少跑路也能办成事。始终把服务企业作为政府履职的重中之重，建立巩固“双千”活动成果长效机制，走进一线为企业帮办、陪办、代办各类事项, 用政府的高效率带来企业高效益、赢得群众好口碑。

建设阳光法治政府。坚持把依法行政贯穿到政府工作的各个领域、各个层面、各个环节，用法治思维和方式履职尽责。建立行政权力清单动态管理机制，做到行政权力进目录、目录之外无特权。强化政府依法行政、企业合法经营、市民守法自律，营造崇尚法治的良好氛围。自觉接受人大法律监督和政协民主监督，广泛听取各民主党派和社会各界人士的意见建议。严格执行重大事项公示、社会听证和政府法律顾问等制度，规范行政决策程序。落实重大决策终身追究和责任倒查制度，健全行政执法自由裁量权基准制度，做到公正文明执法。

建设清正廉洁政府。落实全面从严治党责任，严守《准则》和《条例》，把纪律和规矩挺在前面。坚持源头预防，突出抓早抓小，深化拓展百姓问政、政风行风测评等作风建设载体，切实消除“中梗阻”，驰而不息纠正“四风”。深化重点领域、重大民生项目信息公开，加大财政资金分配、重大项目审批、重大物资采购招标等领域审计力度，强化公共资源交易监管。严肃查处各类违纪违法案件和发生在群众身边的腐败问题，对不作为、慢作为、乱作为的严肃问责，营造风清气正的干事创业环境。

各位代表，立足新起点，展望新征程，我们深感使命重大，责任在肩。让我们在市委的正确领导下，紧紧团结和依靠全市人民，不忘初心，继续前进，用激情绘就蓝图，用实干成就梦想，为决胜全面小康、建设最具幸福感城市做出新的更大贡献，以优异成绩迎接党的十九大胜利召开！
